# Nurses’ perception about posttraumatic growth (PTG) after natural disasters

**DOI:** 10.1186/s12919-020-00199-9

**Published:** 2020-12-08

**Authors:** Eriyono Budi Wijoyo, Herni Susanti, Ria Utami Panjaitan, Arcellia Farosyah Putri

**Affiliations:** 1 Magister of Nursing, Faculty of Nursing, Universitas Indonesia, Depok, Indonesia; 2 Mental Health Nursing Department, Universitas Muhammadiyah Tangerang, Tangerang, Indonesia; 3 Mental Health Nursing Department, Faculty of Nursing, Universitas Indonesia, Depok, Indonesia; 4 Indonesian Emergency and Disaster Nurses Association, Jakarta, Indonesia; 5 School of Health in Social Science, Nursing Studies, University of Edinburgh, Edinburgh, UK

**Keywords:** Nurse perceptions, Posttraumatic growth, Natural disasters

## Abstract

**Background:**

Natural disasters have become the most common, profound, and universal type of catastrophes over decades. Natural disasters can lead to both negative and positive impacts on survivors. Nurses have an important role in fostering posttraumatic growth (PTG) as a positive psychological adjustment amongst the survivors. However, nurses may have different perceptions of their roles in PTG. Such differences may result in various approaches in supporting PTG as best as possible. Therefore, nurses’ perception regarding PTG needs to be explored.

**Method:**

This study used a descriptive qualitative approach. A total of fourteen nurse participants were included across five different cities in Indonesia, including Jakarta, Bogor, Depok, Tangerang, and Bekasi. Data were collected through in-depth interviews and analyzed with a thematic method.

**Results:**

The study revealed three themes, as follows (1) PTG is a new concept for nurses, (2) PTG is a condition that needs to be sought by volunteers, and (3) PTG means human-God and human-human positive relationships.

**Conclusion:**

The study highlights the importance of improving nurses’ understanding regarding PTG so that they can deliver appropriate strategies or interventions to support survivors in gaining positive changes after experiencing a natural disaster. The study recommends that knowledge and skills related to PTG should be introduced in undergraduate nursing program.

## Background

Natural disasters when strike, influence individuals, families, communities, and the environment. The impact of such disasters can be material, physical or psychological loss [1]. A post-traumatic stress disorder (PTSD) has been acknowledged as a common concept, referring to long-term negative psychological impacts of natural disasters [2–4]. However, the impact of traumatic events such as natural disasters can be perceived more positively in the form of posttraumatic growth (PTG) [5, 6]. Tedeschi and Calhoun introduced the notion of PTG to describe enduring positive changes in response to any traumatic event, including natural disasters [7]. Clearly, the consequences of natural disasters could be explained as two sides of the same coin: PTSD involves negative responses, while PTG involves positive responses amongst the survivors [8, 9].

Research around PTG after natural disasters in Indonesia is limited. Two studies have shown that PTG existed among survivors of volcanic eruptions, earthquake, tsunami, and liquefaction [5, 6]. Unfortunately, there is no study to explore nurses’ perception of PTG in the country. As a result, there may be a lack of understanding on how nurses can support PTG of the survivors. Nurses as the biggest frontline health providers need to know of how to foster PTG so they can help to maintain survivors’ resilience and nurture positive thoughts, feelings, and actions even before the occurrence of a disaster [9, 10]. The current investigation was aimed to explore nurses’ perception of PTG after natural disasters in Indonesia.

## Methods

This research employed a qualitative descriptive approach. A qualitative approach is an appropriate method to produce rich and deep data from an exploration of perceptions or perspectives of participants [11]. The research design is in accordance with the research question in order to achieve an understanding of how nurses have comprehended about PTG: What is the perception of Indonesian nurses about PTG? The study involved participants worked as disaster nurses from five adjacent cities, including Jakarta, Bogor, Depok, Tangerang, and Bekasi. These nurses were frequently deployed in a-wide-range areas of natural disasters in Indonesia.

The researchers used a purposive sampling method in which the selection of the participants should correspond to the objective of the study to obtain the best information about the research problems being studied [12]. The inclusion criteria were nurses who (a) lived, and their workplace was in Jakarta, Bogor, Depok, Tangerang, or Bekasi, (b) had a minimal degree of diploma in nursing, and (c) had experiences in responding to natural disasters.

Prior to the interviews, the main researcher [EBW] contacted prospective participants based on information from gatekeepers. The initial contact was carried out face-to-face to provide information about the study. Then, the potential participants were given at least 24 h to decide whether they wanted to participate or not. Next, the researcher contacted the participant to ask for the decision and scheduled an interview if they wished to participate in the study.

The main researcher collected the whole data through in-depth interviews. An interview guideline was prepared based on a review of literature relevant to the research topic, and consultations to experienced researchers [HS and RUP]. The interviews were conducted for 45–60 min and located either at participants’ home or workplace based on their preference. The data collection was conducted in 3 months, from February to April 2019. The process of data collection was terminated after saturation had been achieved.

Data were analyzed thematically that aimed to identify, scrutinize, and report patterns as themes [13]. During the analysis process, all research members involved in the theme generation and interpretation. The trustworthiness of this study was maintained through the peer debriefing by working together with all members. The supervisors [HS, RUP] and an expert in disaster nursing [AFP] examined and provided feedback of the transcripts, interpretation, and results. In addition, the dependability of the study was accomplished by an audit trail when the main researcher discussed with the supervisors and the disaster expert around data collection and analysis. Regular meetings with the supervisors were helpful in revealing the main researcher’s bias, assumptions, and data misinterpretation.

## Results

There was a total of fourteen nurses who participated in this research. The youngest was 21 years old, and the oldest was 56 years old with the equal proportion between female and male nurses. They have a various educational nursing degree, including Diploma, Ners (Bachelor of Nursing), Master, and Mental Health Specialist (Table 1).

**Table 1:** Participants on this research (*n* = 14)

Code	Age	Occupation	Sex	Educational degree	Suku	Experience
P01	27	Postgraduate Student	Male	Ners	Bima	As volunteer in earthquake Lombok (August 14–20, 2018).
P02	39	PNS	Female	Ners	Java	As volunteer in earthquake Pariaman, 2008
P03	32	PNS	Female	Master	Dayak	As volunteer in earthquake Lombok (August 14–20, 2018).
P04	27	Lecturer	Female	Ners	Java	As volunteer in earthquake and tsunami Palu (Oct 22–24, 2018).
P05	30	Nurse	Female	Ners	Padang	As volunteer in eruption Merapi mt. Jogjajarta 2010
P06	38	PNS	Male	Master	Java	1. As volunteer in flood disaster, Aceh 2008; flood disaster, Pangkalan Brandan; earthquake, Padang
P07	33	Lecturer	Male	Master	Wakatobi	As volunteer in earthquake disaster Lombok (August, 27–312,018)
P08	24	Swasta	Male	Ners	Java	As volunteer in earthquake disaster, likuivasi Donggala, Sigi and Palu 2018.
P09	21	Swasta	Female	Diploma	Padang	As volunteer in earthquake disaster, likuivasi Donggala, Sigi and Palu 2018.
P10	29	Military	Male	Diploma-III	Palembang	As volunteer in earthquake disaster Pidi Jaya, tsunami Banten (2018)
P11	32	Military	Male	Diploma-III	Java	As volunteer in flood disaster, Jakarta (2014), earthquake Lombok 2018
P12	56	Lecturer	Female	Master	Sunda	As volunteer in flood disaster, Situ Gintung (2009), Jakarta (2014)
P13	30	Lecturer	Female	Mental Health specialist	Betawi	As volunteer in flood Situ Gintung (2009)
P14	31	Nurses	Male	Ners	Sunda	As volunteer in tsunami, Banten (2018) and earthquake in Lombok.

This research identified three themes regarding the description of nurses’ perceptions on PTG after natural disasters which will be presented in sequence following the identification of the themes (Table 2).

**Table 2:** Themes and Sub-themes

Themes	Sub-themes
1. PTG is a new concept for nurses	They are volunteers who are new to this concept
	Volunteers who are more exposed to the PTSD concept
2. PTG is a condition that needs to be sought by volunteers	The assistance to be delivered by volunteers
	The strategies to be delivered to return the community into their original life
	Training to be delivered to anticipate future disaster
3. PTG means human-God and human-human positive relationships	The process of approaching to God
	Togetherness in living after natural disasters

### Theme 1: PTG is a new concept for nurses

The current theme can be described into two categories: nurses are new about the PTG concept, and nurses who are more exposed to the PTSD concept.

The first category underlines that most nurses in this study were not familiar with PTG concept, even though they have been working in disaster situations for many years. According to them, the concept is new, and they just heard and knew about it after their involvement in the current research, as conveyed by some participants below:

“*… Honestly, I have just heard about this term for the first time …*” *(P04).*

*“… Mm, I did not know about the theory nor the concept …” (P07).*

In the second category, the participants attempted to explain the reason for the unfamiliarity, that was because they have been exposed by PTSD concept dominantly:

*“… Honestly, I have just heard about this theory [PTG]. What I am familiar with is about posttraumatic stress disorder …” (P05).*

*“… Information about PTG is new to me. What we usually have is the information related to PTSD and the intervention …” (P13).*

### Theme 2: PTG is a condition that needs to be sought by volunteers

This theme could illustrate the nurses’ views about the meaning of PTG in relation to some endeavors for achievement. Here, the whole attempts to be delivered by nurses. It is worth to note that, the nurses under this investigation often assigned themselves as volunteers, to indicate that, even though they served as disaster health workers, but they performed their roles in a hazardous situation by sacrifices including leaving their loved ones. The theme has three categories, namely some assistance to be delivered by volunteers, strategies to be delivered to return the survivors into their original life, and some training to be delivered to anticipate future disasters.

The first category is some assistance to be delivered by volunteers, revealed by at least two participants. These nurses emphasized that PTG is a condition in that volunteers need to strive for. Some of the attempts included providing information related to the process of PTG, and psychological support to achieve the state of growth. The participants expressed the ideas in this way:

*“… I think PTG is quite good for some people as it can increase their resilience, their endurance, and their acceptance process, so it needs to be informed to the disaster survivors …” (P13).*

*“… Yes, it [the psychological support] could help a lot, especially related to the survivors*’ *psychological aspect …” (P02).*

The second category is strategies for survivors to return to their original life. According to some participants, PTG is how to bring back people to their life before being affected by natural disasters, and this need professional help as presented below:

*“… In my opinion, posttraumatic growth is about how the victims can go back to their original life … how is our strategy to return the victim to their normal life and condition without prolonged trauma”* (P06).

*“… The special team need to work in terms of mental health, trauma healing, and whatever the name is … to bring back their [survivors’]life(P08).*

The third category is some training to be delivered to anticipate following disasters. The participants indicated that when talking about PTG; it is not only a notion for health workers to be expertise but also a duty for professionals to train the survivors, so they can understand its concept thus anticipate the trauma following a natural disaster. These were stated as follows:

*“… Only a few people think that it may direct to a positive impact”(P03).*

*“it is more like trying to develop certain anticipation within them … related to the disasters, that they are prepared for this and that …”* (P12).

### Theme 3: PTG means human-god and human-human positive relationships

There are two categories that illuminate the perspectives of nurses about PTG, which is directed to a positive relationship between survivors and God, and the subsequence of this connection to humans: The process of approaching to God, and togetherness in living after natural disasters.

The first category, the process of approaching God, is revealed by participants with code P04, P07 and P14. These nurses suggested that a positive view about catastrophe could be observed when survivors get closer to God, affirmed by their engagement in religious activities such as praying together at mosques. In addition, PTG can be characterized when survivors view a disaster as a gift for God’s opportunity to keep them alive. Sometimes, positive outlook about a natural disaster means that humans have a momentous episode to reflect their sentiment to God, before and after a disaster hit. These were expressed as follows:

*“… I also frequently saw them praying together in the mosque, at that time when we arrived at Sembalun [an earthquake area] during the sunset prayer, there were many of them prayed …” (P07).*

*“… the opportunity to live after the disaster is a gift from God; they can [think like this] because of PTG …” (P04).*

*“… The disaster can become a channel... a factor for changes in a clan, a region, a community, in that that we hope the negative feeling about God will turn to be more positive because we are afraid of other disasters to come …” (P14).*

The second category is togetherness in living after a natural disaster. The current category can be considered as the following characteristic of survivors who have achieved PTG after their connection with God has been reached satisfactorily. These participants anticipated that some survivors had acknowledged positive impacts of a disaster, as shown by their togetherness with families, neighbors, and those in shelters; and they help each other after the strike. These were stated as follows:

*“… they live their life after the earthquake together … with their families … their communities …” (P01).*

*“… they spread positive energy to others so that they can rise from the downturn …” (P08).*

*“… The survivors think a lot about their life after a disaster, and automatically I can see that the trauma turns them from being individuals to togetherness …” (P09).*

## Discussion

### Theme 1: PTG is a new concept for nurses

PTG is a form of positive change from past experience that occurs as a result of struggling with traumatic events such as disaster, chronic illness, or rape [10, 14–16]. These changes may include change of perceptions, feelings, and behavior towards a more positive notion after a traumatic incident [9, 10]. However, PTG is considered as a new concept for participants in our study. They are more exposed to the concept and management of post-traumatic stress disorder (PTSD). The reason is maybe the concept of PTG is just developed by psychologists Richard Tedeschi and Lawrence Calhoun in mid-1996. They stated that people who survived psychological struggles after experiencing difficulties could see positive growth afterwards. This concept sees the development of new understandings of oneself, the world in which they live, how to relate to others, the kind of future they may have, and a better understanding of how to live their life after a traumatic incident [15, 17].

Participant P04 and P07 said that they did not know this concept and just heard it for the first time as they were involved in the current study. While taking formal education in nursing education, participants have not gained knowledge about this concept. According to the Indonesian Nurses Education Core Curriculum in 2015, through formal undergraduate nursing education, knowledge about a disaster, in general, have been delivered to nursing students. Subjects related to disaster are given in semester 7 (seven) and cover discussing concepts, types, classifications, characteristics of disasters, and the impact of disasters on health [18]. However, a specific discussion about PTG is absence. Likewise, in nursing diploma education, the subject about the disaster has been included in the education curriculum, which is summarized in emergency nursing courses and disaster preparedness management for two credits, but no information provided concerning PTG concept. The core curriculum of The Association of Indonesian Nurse Education Center (AINEC) for bachelor degree in nursing and Association of Indonesian Vocational Nurse Education Center (AIVNEC) for vocational in nursing is being processed in 2014 for Vocational Nursing and Bachelor Nursing [19]. This was being socialized and implemented by educational institutions in early 2016.

Participant P05 and P13 stated that nurses had just heard about the concept of PTG and were more exposed to the concept of PTSD. Research related to PTSD is more than PTG, so nurses are rarely exposed to the PTG concept [15, 20]. This is similar to what P13 participant said that participants were more exposed to the concept of PTSD and other negative syndromes so that the intervention also led to PTSD rather than PTG. In addition, P05 participant also said that during education, discussions related to PTG were not offered and those that were more frequently raised in terms of diagnoses for disorders were PTSD in patients with trauma. For instance, unlike PTG, diagnosis of PTSD is included in the Diagnostic and Statistical Manual of Mental Disorders (DSM-5) as exposure to certain types of trauma, such as death, serious injury, and rape victims (both those who see and experience directly) which produce symptoms of negative changes in the victim’s beliefs and feelings [21, 22]. In nursing, there are nursing diagnoses related to trauma conditions, namely post-traumatic syndrome. The definition of the post-traumatic syndrome is a continuous maladaptive response to traumatic incidents. In the North American Nursing Diagnosis Association International (NANDA-I) nursing diagnosis 2018–2020, there is no diagnosis for adaptive response to traumatic events, such as PTG, so nurses who study the diagnosis are not forthcoming towards an adaptive response after experiencing trauma, one of which due to natural disasters [23, 24]. This proves that nurses are more exposed and learn more about PTSD than PTG. This situation highlighted that the concept of PTG should be introduced even in early nursing education.

### Theme 2: PTG is a condition that needs to be sought by volunteers

Natural disasters, when strike, are commonly unexpected and sudden. Thus, survivors may need time to effectively cope with such events. Nevertheless, natural disasters can lead to Posttraumatic Growth (PTG) [8, 9]. The posttraumatic growth is a positive change from an incidence of trauma, one of which is a natural disaster. Here, a change refers to a survivor’s adjustment from experiencing a traumatic event to challenge one’s views and perspectives and result in the process of coping, reappraisal an adaption to the situation [5].

The current research suggests that survivors cannot achieve PTG straightforwardly, as indicated in the second theme: PTG is a condition that needs to be sought by volunteers. This finding is in line with some studies in similar fields, which suggest that a positive-growing-outlook from a traumatic event is achievable with supports from health professionals, including nurses. For example, a study by Prati and Pietrantoni discovered that PTG was achieved by survivors through the help of health workers who nurture optimism, provide social support, and teach coping strategy to survivors [15].

In the current study, participants P02, P03, P05, P06, P09, P10, P11 and P14 have collaborated with related parties such as non-health volunteers, military, national and international Non-Government Organizations (NGOs) in formulating a plan for survivors to help them recover from traumatic events such as disasters. Good communication between nurses and other teams can accelerate the recovery phase of disaster survivors [25, 26]. Participants also highlighted that their main goal in recovery phase was to help survivors returning to their normal life and prevent prolonged trauma. Such activities, which were carried out by participants, according to Mikal-Flynn, can be categorized as strategies to guide individuals towards PTG [27].

Nurses who are deployed to the disaster area are expected to have skills and knowledge related to psychological support. These skills include the ability to conduct mental health screening after a disaster, refer survivors to healthcare services, respond quickly and accurately in helping victims, deliver post-disaster mental care, collect evaluation data related to post-disaster responses, and perform Psychology First Aid (PFA) [20, 28]. Moreover, nurses must be more aware of their unique role in encouraging PTG among disaster survivors. In the current study, nurses who delivered the psychological support were participant P02, P04, P07, P08, P10, P11, P12 and P13. These nurses had received Psychology First Aid (PFA) training so they could carry out post-disaster assessments and provided support for survivors while they were staying at refugee camps. Participant P01, P09 and P14 also gave psychosocial support at field hospitals and health clinics.

### Theme 3: PTG means human-god and human-human positive relationships

Positive changes in the relationship of God-human and human-human are included in the concept of posttraumatic growth theory after traumatic incidents [10]. Participant P04, P07 and P14 stated that the whole community was getting closer to God. They prayed together and felt thankful that they are still alive. Other studies highlight that religion and spirituality can improve one’s ability to cope with crisis [25, 26, 29]. Praying to God is believed to bring peace to disaster survivors and help them go through all difficulties [30].

Moreover, studies highlighted that, within PTG, relationships with others become closer, more intimate, and meaningful [7, 9]. This is in line with the statements of participant P01, P08, P09, P10 and P11 that there were positive changes of the relationship such as togetherness within the community and willingness to help each other were increased. Having positive relationships is strong social support that can help someone cope with trauma, the stronger the social support, the better the acceptance [4, 31]. Some studies also revealed that social support is a strong influential factor in the PTG process [15, 26]. Social support can increase self-resilience that can minimize PTSD and foster PTG among earthquake survivors [8].

Despite the strength of this study which explored a scarce topic surrounding disaster phenomenon from the perspective of nurses in Indonesia, the participants have not represented care providers across the country. However, the whole nurses involved in the study have experiences in providing services in a-wide-range natural disasters occurred in many places of the nation.

## Conclusion

The study discovered three main themes that indicate the views of Indonesian nurses about PTG. These include a description that PTG has been perceived as a new concept amongst nurses [Theme 1]. However, some attributes of PTG are highlighted, including PTG requires efforts from volunteers [Theme 2] and can bring positive human-God and human-human relationships [Theme 3]. The study highlights the importance of improving nurses’ understanding of PTG so that they can deliver appropriate strategies or interventions to support disaster survivors moving towards more positive changes. Education and training for nurses on how to facilitate disaster survivors to achieve PTG are recommended.

## Data Availability

Interview transcript are available from related authors at reasonable request.

## Abbreviations

AFP: Arcellia Farosyah Putri
AINEC: Association of Indonesian Nurse Education Center
AIVNEC: Association of Indonesian Vocational Nurse Education Center
DSM-5: Diagnostic and Statistical Manual of Mental Disorders
EBW: Eriyono Budi Wijoyo
HS: Herni Susanti
Jabodetabek: Jakarta, Bogor, Depok, Tangerang and Bekasi
NANDA-I: North American Nursing Diagnosis Association-International
NGOs: Non-Government Organizations
PFA: Psychology First Aid
PTG: Posttraumatic Growth
PTSD: Post-traumatic stress disorder
RUP: Ria Utami Panjaitan
TIU NNA: Technical Implementation Unit National Narcotics Agency
